# Continued range expansion of *Aedes albopictus* (Diptera: Culicidae) in Iowa, United States

**DOI:** 10.1093/jme/tjag052

**Published:** 2026-04-03

**Authors:** Christopher H Lee, David R Hall, Ryan C Smith

**Affiliations:** Department of Plant Pathology, Entomology and Microbiology, Iowa State University, Ames, IA, USA; Department of Plant Pathology, Entomology and Microbiology, Iowa State University, Ames, IA, USA; Department of Plant Pathology, Entomology and Microbiology, Iowa State University, Ames, IA, USA

**Keywords:** *Aedes* albopictus, invasive species, mosquito surveillance, vector ecology

## Abstract

The mosquito *Aedes albopictus* (Skuse) (Diptera: Culicidae) serves as a competent vector of several arboviruses, including dengue, chikungunya, and Zika viruses, and is highly invasive, spreading to every continent except Antarctica. With the emergence of Zika virus in the Americas in 2015 and 2016, targeted surveillance efforts for invasive *Aedes* species were initiated in Iowa, documenting the detection and establishment of *Ae. albopictus* in 3 Iowa counties over a 5-yer period (2016 to 2020). Herein, we provide an update on the abundance of *Ae. albopictus* based on our continued surveillance efforts over the past 5 yr (2021 to 2025) across 19 Iowa counties, further documenting its spatial distribution and temporal abundance in the state to at least 11 counties. Highlighting trapping efforts in 2 Iowa counties with previous or recently detected populations of *Ae. albopictus*, we provide a detailed examination of their increased detections in these locations, offering new insight into their continued spread in the state to locations that were previously negative for *Ae. albopictus*. Together, these data provide further evidence for the continuing range expansion of *Ae. albopictus* in Iowa, which serves as its current northern range in the Upper Midwest. These findings have broad implications for the future spread of this invasive mosquito species in the United States and its potential impacts on public health.

## Introduction

*Aedes albopictus* is a highly invasive mosquito species and efficient vector of several arboviruses that pose a substantial burden to global public health. First introduced to the United States in 1985 (Sprenger and Wuithiranyagool 1986), *Ae. albopictus* has continued its spread to at least 26 states and established itself in environments previously thought to be inhospitable for further expansion due to climate and questionable overwintering success (Hahn et al. 2016, 2017, Yee 2016).

Prior to 2016, *Ae. albopictus* was only sporadically detected in Iowa, suggesting that these detections were isolated and likely unsuccessful introduction events (Hahn et al. 2016, Kovach and Smith 2018). Following the emergence of Zika virus in the Americas in 2015 to 2016, targeted surveillance was implemented in Iowa to monitor invasive *Aedes* species, leading to the detection of *Ae. albopictus* in 3 counties in 2017 (Hall et al. 2022). These populations were considered established based on multi-year abundance data (2017 to 2020) and genetic haplotyping (Hall et al. 2022), despite previous suggestions that Iowa would be unlikely to be suitable for *Ae. albopictus* (Johnson et al. 2017).

Herein, we update the distribution and abundance of *Ae. albopictus* in Iowa over the last 5 yr (2021 to 2025) through continued surveillance efforts, providing evidence of the continued expansion and establishment of *Ae. albopictus* in additional surveyed counties. We provide examples of 2 Iowa counties with differing histories of *Ae. albopictus* establishment, demonstrating their increased abundance and intra-county spread to previously negative sites. Given that *Ae. albopictus* is a competent vector of several arboviruses, its growing presence in Iowa raises concerns for its further expansion in the Upper Midwest and its associated public health implications.

## Materials and Methods

### Mosquito Collections

Mosquito trapping was conducted across Iowa from approximately May to October between 2021 and 2025 by Iowa State University personnel in collaboration with county public health agencies and private citizens. Adult mosquitoes were collected from 201 total traps located across 144 trap sites in 19 counties (Supplementary Table S1). *Ae. albopictus* specimens were predominantly collected as part of our targeted *Aedes* surveillance program using Biogents BG-Sentinel traps (BG) and Biogents Gravid Aedes traps (GAT), although some samples were collected incidentally as part of our West Nile virus (WNV) surveillance, including New Jersey Light Traps (NJLT), CDC Light Traps (CDC), and Frommer Updraft Gravid Traps (Gravid). Male and female mosquito samples were identified according to morphological characteristics (Darsie and Ward 2005), with the presence/absence of *Ae. albopictus* assessed for all trap types in each county.

### Data Analysis

Raw mosquito data were processed to determine the total number of mosquitoes collected by site and by year for each species. Mosquito abundance data were normalized using the trap index (number of mosquitoes per trap night) or as the proportion of *Ae. albopictus* (of the total mosquitoes) for each site. To examine the abundance and spread in Polk and Fremont counties, trapping data were examined over the entire study period, with the exception that only sites in Polk County where *Ae. albopictus* were previously negative before 2021 and with ≥3 yr of data were included. Trap indices and the proportion of *Ae. albopictus* (of total mosquitoes) were plotted using GraphPad Prism software (San Diego, California). Data are for descriptive purposes only, lacking statistical power for robust analysis.

### Geospatial Analysis

GPS coordinates of *Ae. albopictus* positive and negative trap sites from all surveyed counties were combined with an Iowa county boundary shapefile (Iowa Geospatial Data Clearinghouse; https://geodata.iowa.gov/) to create distribution maps for the presence/absence of *Ae. albopictus*. GPS coordinates of trap locations were referenced to the WGS84 geographic coordinate system (EPSG : 4326) in ArcGIS Pro 3.4.3.

## Results

### Increased Abundance and Distribution of *Ae. albopictus* Across Iowa

**Fig. 1.**
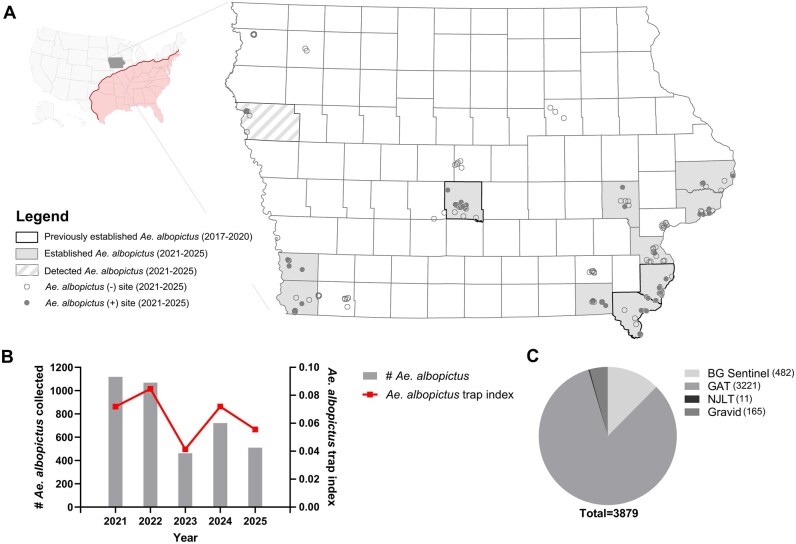
Overview of mosquito trapping locations and abundance of *Ae. albopictus* in Iowa (2021 to 2025). A) Mosquito surveillance efforts were performed in 19 Iowa counties from 2021 to 2025, with *Ae. albopictus* either detected or established in 11 counties. The inset map displays the location of Iowa in the United States, with the red line and shaded red area denoting likely habitat for *Ae. albopictus* as defined by Hahn et al. (2017). *Ae. albopictus* collected from 2021 to 2025 are displayed as raw and normalized (trap index) numbers by year (B) or by trap type (C).

Previously, surveillance data from 2016 to 2020 confirm the detection and establishment of *Ae. albopictus* in 3 Iowa counties (Hall et al. 2022). Herein, ongoing surveillance from 2021 to 2025 demonstrates the detection of *Ae. albopictus* in a total of 11 Iowa counties (Fig. 1A). This includes the 3 counties previously positive for *Ae. albopictus* (Des Moines, Lee, and Polk; Hall et al. 2022), as well as 8 additional counties (Fig. 1A). *Ae. albopictus* was detected in these locations at multiple trapping sites or in multiple years, supporting the presence of locally established *Ae. albopictus* populations (Fig. 1A). However, 1 county (Woodbury) is an exception, where only a single *Ae. albopictus* was detected in 2022 despite multiple years of trapping, suggesting that its detection was likely an isolated and unsuccessful introduction event (Fig. 1A**, Table S2**). A compendium of surveyed counties and *Ae. albopictus* detections from 2016 to 2025 demonstrates the expansion of this species across Iowa (Supplementary Fig. S1).

Trapping efforts from 2021 to 2025 yielded a total of 3,879 *Ae. albopictus* from all trap types, comprising 1.04% of all mosquitoes collected. Raw and normalized (trap index) numbers for *Ae. albopictus* varied between years, with lows for both metrics in 2023 (Fig. 1B). Declines in *Ae. albopictus* numbers from 2021 to 2025 correspond with reduced trapping efforts during this study period, while trap indices remain consistent (Fig. 1B, Supplementary Table S1). *Ae. albopictus* samples were primarily collected using GATs, with 3,221 *Ae. albopictus* collected across 121 traps and representing ∼83% of the total *Ae. albopictus* yields (Fig. 1C). The remaining samples were collected from BGs, Gravid traps, and NJLTs (Fig. 1C). GATs displayed the highest yields, with a trap index of 0.15 *Ae. albopictus* collected per trap night (Supplementary Table S3).

### Analysis of *Ae. albopictus* Expansion in Two Iowa Counties

**Fig. 2.**
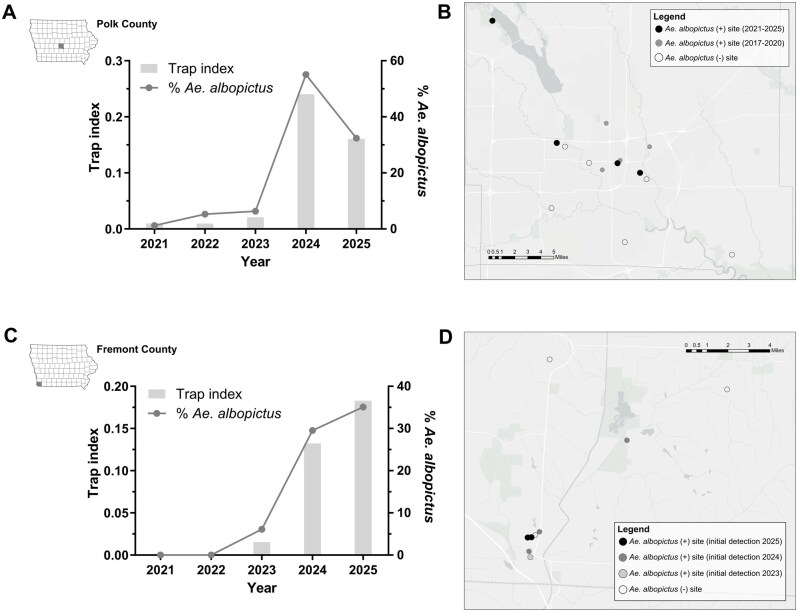
Spread and expansion of *Ae. albopictus* in 2 Iowa counties. A) Annual trap index (bar) and percentage of *Ae. albopictus* in Polk County (inset map) from 2021 to 2025, with trapping site locations in Polk County displayed spatially in B). Black circles represent sites with recent detections of *Ae. albopictus* from 2021 to 2025, while previously positive sites from 2017 to 2020 are shown in gray. C) Annual trap index (bar) and percentage of *Ae. albopictus* in Fremont County (inset map) from 2021 to 2025, with trapping site locations in Fremont County displayed spatially in (D). Shaded circles denote sites according to the respective year in which *Ae. albopictus* was first detected. The presentation of data is meant for qualitative purposes only due to limitations in sample size that limit statistical power and subsequent analysis.

Using the increased yield and abundance of deployed GATs, we examined the spread of *Ae. albopictus* in Iowa by focusing on Polk and Fremont counties. In Polk County, where *Ae. albopictus* have been consistently detected since 2017 (Hall et al. 2022), we observe an increase in the trap index and proportion of *Ae. albopictus* collected from 2021 to 2025 (Fig. 2A). While we previously identified 4 *Ae. albopictus*-positive sites in Polk County from 2017 to 2020, all positive locations were within 3 miles of a tire recycling facility that was likely the initial site of introduction (Hall et al. 2022). From 2021 to 2025, the number of *Ae. albopictus*-positive sites increased to eight, including detections at 4 previously negative sites that extend more than 15 miles from the putative initial introduction site (Fig. 2B, Supplementary Table S4).

Previous efforts dating back to 2016 failed to detect *Ae. albopictus* in Fremont County (Kovach and Smith 2018, Hall et al. 2022), yet in 2023 *Ae. albopictus* was detected for the first time, representing a unique opportunity to monitor its further local spread. From 2023 to 2025, *Ae. albopictus* displayed a steady increase in abundance and the relative proportion of mosquitoes collected (Fig. 2C). Following the collection of *Ae. albopictus* at a single site in 2023, *Ae. albopictus* were collected from a total of 7 sites by 2025 (Fig. 2D, Supplementary Table S4), including sites more than 7 miles from the initial site of detection in Fremont County.

## Discussion

Following the detection of *Ae. albopictus* in 3 Iowa counties in 2017 (Hall et al. 2022), a primary objective of our surveillance efforts has been to monitor the continued spread of *Ae. albopictus* in the state. Using data collected from 2021 to 2025, we provide evidence for the detection of *Ae. albopictus* in 11 Iowa counties, with established *Ae. albopictus* populations are likely in 10 of these counties. Given this increase in *Ae. albopictus*-positive counties from 3 to 11 in just 5 yr (2021 to 2025), we believe that *Ae. albopictus* has not only become established in Iowa, but also has thrived as it continues its further expansion across the state and its northern boundary in the Upper Midwest.

With *Ae. albopictus* and other invasive mosquito species continuing to extend into new areas, an outstanding question remains whether a given mosquito species has become established in a given location or if detections are due to introduction from external sources (such as the tire trade or intra/interstate commerce). Unlike tick surveillance, where clear criteria have been established by the CDC to determine the establishment of tick species (CDC 2020), similar guidelines have not been created for the establishment of a mosquito species. As a result, we propose that several factors be taken into consideration, such as consistent or increasing annual detections without a source of introduction; early seasonal abundance; detection at multiple locations; evidence of localized spread; and, where possible, the identification of overwintering eggs or genetic haplotype information to support persistence between years. While these are not absolute, we believe that multiple lines of evidence are essential when evaluating the potential establishment of invasive mosquito populations. Using these criteria as a general framework in present and past studies (Hall et al. 2022), we report established populations of *Ae. albopictus* in 10 counties, and similarly use these conditions to dismiss the detection in Woodbury County as a likely isolated introduction event. However, further surveillance is required to fully delineate the presence of *Ae. albopictus* in Iowa, with the potential that *Ae. albopictus* may have spread into other counties not currently monitored through our surveillance program.

As further evidence of the spread of *Ae. albopictus* in Iowa, we provide data from Polk and Fremont counties, which, respectively, were previously established (Hall et al. 2022) or where *Ae. albopictus* were detected. Both counties display an increased abundance of *Ae. albopictus* and significant intra-county dispersal from potential sources of introduction to previous *Ae. albopictus*-negative sites from past surveillance seasons. This provides strong evidence that *Ae. albopictus* is expanding to new locations in Iowa, potentially aided by human-related means of transportation. While the origin of *Ae. albopictus* in Fremont County is unknown, its close proximity and shared border with Missouri, where established populations of *Ae. albopictus* have been previously reported (Hahn et al. 2016, 2017, Claborn et al. 2018), is the most likely source of introduction.

Our data also demonstrate the importance of long-term and sustained surveillance efforts. Both Fremont and Van Buren County, where *Ae. albopictus* was, respectively, first detected in 2023 and 2024, have had continuous surveillance efforts since 2016 (Kovach and Smith 2018, Hall et al. 2022). Other *Ae. albopictus-*positive counties, such as Clinton, Johnson, and Louisa, have had continuous surveillance since 2017 (Hall et al. 2022). As a result, the long-term data for these counties provides an essential baseline for mosquito populations before the introduction of *Ae. albopictus*, offering a unique perspective to follow the active invasion of this mosquito species in these locations.

With limitations in the capacity of our surveillance program to cover all 99 Iowa counties, this study also demonstrates the importance of citizen science for the detection of *Ae. albopictus* in non-surveyed counties. This was evident in 2022, when we received reports from 2 separate individuals regarding *Ae. albopictus* activity at or near their residences in Scott County. Both private citizens were provided GATs to confirm the presence of *Ae. albopictus* at their residences, providing cause for a more widespread deployment of GATs across Scott County by Iowa State personnel in 2022 and 2023 to further investigate the species’ abundance and ultimately its establishment in the county. While isolated detections of *Ae. albopictus* had been previously reported in Scott County, active surveillance efforts through 2020 had not detected *Ae. albopictus* (Hall et al. 2022), highlighting the importance of citizen science to enhance mosquito surveillance activities.

In summary, our study provides evidence of the ever-expanding range of *Ae. albopictus* in Iowa, demonstrated by its increased abundance and spread in previously established locations, as well as its detection in several new counties. With climate change and increasing urbanization further driving the range expansion of *Ae. albopictus*, as well as other mosquito vectors, risks of mosquito-borne disease transmission are expected to coincidentally rise. In addition, with recent reports of local dengue transmission in Florida, California, and Texas in 2024 (Centers for Disease Control and Prevention 2025), these instances highlight the growing potential of arboviral outbreaks in the United States. Therefore, the continued monitoring of *Ae. albopictus* in Iowa and other states is essential, providing critical information to guide public health departments in developing targeted surveillance and control strategies aimed at preventing future arboviral transmission.

## Supplementary Material

tjag052_Supplementary_Data
