# Supplementary material for: Continued range expansion of Aedes albopictus (Diptera: Culicidae) in Iowa, United States
Source: J Med Entomol. 2026 Apr 3;63(2):tjag052. doi: 10.1093/jme/tjag052 (PMC13047283; doi:10.1093/jme/tjag052)
Supplement: tjag052_Supplementary_Data [file tjag052_supplementary_data.zip › Figure S1.docx]

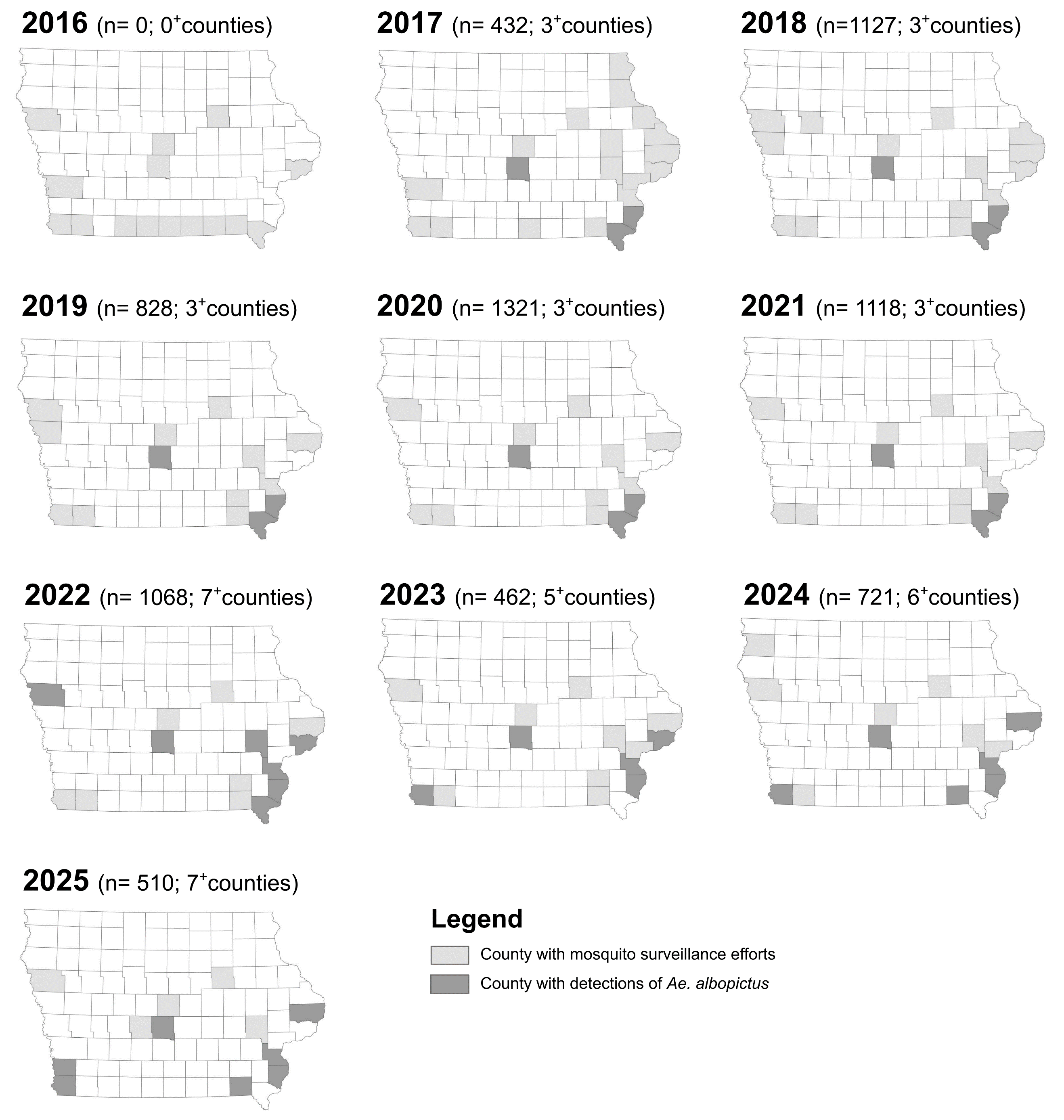


**Fig. S1. Annual county-level detections of *Ae. albopictus* in Iowa (2016-2025).** For each year from 2016 to 2025, each annual map displays the counties where surveillance was performed (light grey), counties where *Ae. albopictus* were detected (dark grey), and the remaining counties where no surveillances activities were performed (white). Data are summarized as the total number (n) of *Ae. albopictus* collected and the number of *Ae. albopictus*-positive counties.
