# Supplementary material for: Continued range expansion of Aedes albopictus (Diptera: Culicidae) in Iowa, United States
Source: J Med Entomol. 2026 Apr 3;63(2):tjag052. doi: 10.1093/jme/tjag052 (PMC13047283; doi:10.1093/jme/tjag052)
Supplement: tjag052_Supplementary_Data [file tjag052_supplementary_data.zip › Table S2.docx]

| **Table S2. Total number of *Ae. albopictus* collected in each Iowa county** (2021-2025) | | | | | | | | |
| --- | --- | --- | --- | --- | --- | --- | --- | --- |
|  |  |  |  |  |  |  |  |  |
| **County** | **2021** | **2022** | **2023** | **2024** | **2025** |  |  |  |
| Clinton | 0 | 0 | 0 | 13 | 5 |  |  |  |
| Des Moines | 164 | 336 | 366 | 358 | 220 |  |  |  |
| Fremont | 0 | 0 | 8 | 75 | 94 |  |  |  |
| Johnson | 0 | 2 | 0 | 0 | 0 |  |  |  |
| Lee | 88 | 168 | - | - | - |  |  |  |
| Louisa | 0 | 4 | 3 | 2 | 3 |  |  |  |
| Mills | - | - | - | - | 45 |  |  |  |
| Polk | 866 | 535 | 42 | 272 | 138 |  |  |  |
| Scott | - | 22 | 43 | - | - |  |  |  |
| Van Buren | 0 | 0 | 0 | 1 | 5 |  |  |  |
| Woodbury | 0 | 1 | 0 | 0 | 0 |  |  |  |
| **Total** | **1118** | **1068** | **462** | **721** | **510** |  |  |  |
|  |  |  |  |  |  |  |  |  |
| "-" denotes that mosquito surveillance was not performed | | | | |  |  |  |  |
