# Supplementary material for: Continued range expansion of Aedes albopictus (Diptera: Culicidae) in Iowa, United States
Source: J Med Entomol. 2026 Apr 3;63(2):tjag052. doi: 10.1093/jme/tjag052 (PMC13047283; doi:10.1093/jme/tjag052)
Supplement: tjag052_Supplementary_Data [file tjag052_supplementary_data.zip › Table S3.docx]

| **Table S3. *Ae. albopictus* numbers and trap indices according to trap type** | | | | | | | |
| --- | --- | --- | --- | --- | --- | --- | --- |
|  |  |  |  |  |  |  |  |
| **County*** | **NJLT** *(TI)* | **Gravid** *(TI)* | **CDC** *(TI)* | **BG** *(TI)* | **GAT** *(TI)* | **Total** *(TI)* | **% *Ae. albopictus*** |
| Clinton (2024-2025) | - | - | - | 0 *(0)* | 18 *(0.0156)* | 18 *(0.0131)* | 1.68% |
| Des Moines (2021-2025) | - | - | - | 362 *(0.303)* | 1,082 *(0.2584)* | 1,444 *(0.2683)* | 30.68% |
| Fremont (2023-2025) | - | - | - | 0 *(0)* | 177 *(0.1107)* | 177 *(0.0999)* | 18.85% |
| Johnson (2022-2025) | 0 *(0)* | 0 *(0)* | - | 1 *(0.002)* | 1 *(0.0004)* | 2 *(0.0005)* | 0.02% |
| Lee (2021-2022) | - | - | - | 98 *(0.123)* | 158 *(0.0780)* | 256 *(0.0906)* | 13.25% |
| Louisa (2022-2025) | - | - | - | 6 *(0.014)* | 6 *(0.0028)* | 12 *(0.0046)* | 0.53% |
| Mills (2025) | 0 *(0)* | 4 *(0.017)* | - | 11 *(0.087)* | 30 *(0.2308)* | 45 *(0.0732)* | 6.13% |
| Polk (2021-2025) | 10 *(0.002)* | 161 *(0.037)* | - | - | 1,682 *(0.3805)* | 1,853 *(0.1411)* | 0.56% |
| Scott (2022-2023) | - | - | - | - | 65 *(0.1952)* | 65 *(0.1952)* | 70.65% |
| Van Buren (2024-2025) | - | - | - | 4 *(0.017)* | 2 *(0.0017)* | 6 *(0.0043)* | 0.44% |
| Woodbury (2022-2025) | 1 *(0.001)* | 0 *(0)* | 0 (*0*) | 0 *(0)* | 0 *(0)* | 1 *(0.0002)* | 0.01% |
| **Total** | **11** *(0.001)* | **165** *(0.026)* | **0** *(0)* | **482** *(0.122)* | **3221** *(0.1512)* | **3879** *(0.1007)* | **1.04%** |
|  |  |  |  |  |  |  |  |
| "-" denotes that mosquito surveillance was not performed | | |  |  |  |  |  |
| *County data only include years after *Ae. albopictus* was initially detected, *TI* = trap index | | | | | |  |  |
